# A novel bio-engineering approach to generate an eminent surface-functionalized template for selective detection of female sex pheromone of *Helicoverpa armigera*

**DOI:** 10.1038/srep37355

**Published:** 2016-11-28

**Authors:** Parikshit Moitra, Deepa Bhagat, Rudra Pratap, Santanu Bhattacharya

**Affiliations:** 1 Department of Organic Chemistry, Indian Institute of Science, Bangalore 560 012, India; 2 National Bureau of Agriculturally Insect Resources, P.B. No. 2491, H. A. Farm Post, Bangalore 560 024, India; 3 Centre of Nano Science and Engineering, Indian Institute of Science, Bangalore 560 012, India

## Abstract

Plant pests exert serious effects on food production due to which the global crop yields are reduced by ~20–40 percent per year. Hence to meet the world’s food needs, loses of food due to crop pests must be reduced. Herein the silicon dioxide based MEMS devices are covalently functionalized for robust and efficient optical sensing of the female sex pheromones of the pests like *Helicoverpa armigera* for the first time in literature. The functionalized devices are also capable of selectively measuring the concentration of this pheromone at femtogram level which is much below the concentration of pheromone at the time of pest infestation in an agricultural field. Experiments are also performed in a confined region in the presence of male and female pests and tomato plants which directly mimics the real environmental conditions. Again the reversible use and absolutely trouble free transportation of these pheromone nanosensors heightens their potentials for commercial use. Overall, a novel and unique approach for the selective and reversible sensing of female sex pheromones of certain hazardous pests is reported herein which may be efficiently and economically carried forward from the research laboratory to the agricultural field.

Plant pests seriously affect food production due to which the yields of the global crop are reduced by ~20–40 percent each year, as estimated by the Food and Agricultural Organization (FAO), the UN based International Plant Protection Convention Secretariat^1^. FAO also projects that the world will need to produce more than 60% food to feed the growing world population by 2050. Hence to meet the world’s food needs, loses of food due to crop pests must be reduced significantly. *Helicoverpa armigera* (Hubner) and *Scirphophaga incertuals* (Walker) are two of the most serious pests as they feed on more than 150 crops across the world. Scientists have developed several methods to control these pests during the last few decades, some of which employ the use of trap cropping^2^, biopesticides^3^, transgenic crops^4^,^5^,^6^,^7^ etc. However, the overuse of pesticides or insecticides together with the change in climate introduces severe pest resistance and hence farmers often use their pheromone traps for the management of these pests^8^,^9^. The regular replacement of these traps, however, makes this procedure expensive and the unnecessary exposure of pheromones even in the absence of pests damages the environment by anthropogenic contamination. Efficient use of pheromones and other semiochemicals, which attract and eventually trap the male insects, can be achieved by applying more accurate methods of detection and quantification. But the scope and applications of current pheromone sensors are limited because of the need of portability, longevity, sensitivity and selectivity in a single device. Again, the sensing and quantification of semiochemicals, especially pheromones, are challenging because they are released at very low concentrations into a chemically diverse environment. Hence it is quite a challenging task for the researchers to sense or measure the concentration of the pheromone released by the crop pests by various analytical techniques such as electroantennogram^10^,^11^, single sensillum^12^, pheromone guided mobile robot^13^ and electronic nose^14^ etc. Most of these techniques have common disadvantages of periodic replacement of antennae of insects (as the lifetime of antenna is limited) and maintenance of live culture to get antennae. These factors make these techniques prohibitively expensive and commercially inapplicable. Also these techniques are not user friendly as the farmers need specialized training for the insect identification, rearing and culturing specific insects to get antennae.

Herein, we introduce a novel cost-efficient pheromone sensing based pest detection that uses a microelectromechanical system (MEMS) device^15^,^16^,^17^,^18^,^19^,^20^,^21^,^22^,^23^,^24^,^25^,^26^,^27^,^28^,^29^,^30^ especially designed and functionalized for this purpose. We have developed a MEMS sensor using microcantilevers and fixed-fixed beams as resonant mass sensors for the selective detection of female sex pheromone of *Helicoverpa armigera* (Hubner), lepidopterous pest of cotton, tomato, rice, pigeonpea and chickpea etc., in air. To the best of our knowledge, this is the first study where silicon dioxide based MEMS devices are covalently functionalized to selectively detect the pheromone molecules for specific insects with sensitivity upto femtogram (fg) level. The successful functionalization of the substrate has been verified by various analytical techniques such as atomic force microscopy (AFM), laser doppler vibrometry (LDV), X-ray photoelectron spectroscopy (XPS) and time-of-flight secondary ion mass spectrometry (ToF-SIMS). The relative sensitivity of these sensors was further improved by different functionalization protocols which increased the number of amine functionalities at each anchor site. The detection limit was observed to be 4.06 ± 0.5 fg of pheromone mass attached to the functionalized cantilevers, which is well below the concentration found for the pheromone at the time of infestation as per the OECD Monograph Guidance – Pheromones and Semiochemicals - September 2002^31^. These MEMS sensors not only eliminate the use of bulky, complex, and fragile instruments, but also reduce the cost considerably for large-scale sensor deployment in real field. Further these pheromone sensors show excellent efficacy and stability in the open atmosphere, even during wet atmospheric conditions like rainy season or in peak summer under bright sunshine. This feature together with the trouble-free transportation aspect of this reversible sensor heightens their potential for the commercial use in any season under ambient conditions. In addition, the chemical functionalizations of the devices have photochemical and thermal stability. The functionalized MEMS devices are also capable of estimating the pheromone concentration present in a field or vineyard and thus may help in determining the level of infestation. The recognition of the pheromone molecules even before visual onset may alert the farmers to take necessary actions in a localized manner before crop loss starts to occur. Hence this approach may be efficiently and economically used in agricultural farms to significantly reduce crop losses and the attendant financial losses.

## Results and Discussion

### Fabrication and Functionalization of the MEMS Devices

Herein, the silicon dioxide based surface-micromachined cantilevers and fixed-fixed beams were first fabricated (Fig. S1) and then successfully modified their surface for selective detection of the female sex pheromones of *Helicoverpa armigera* and the like. We specifically targeted the pheromone for efficient pest management as they are species specific and chose the silicon dioxide platform of the devices for the convenience in covalent functionalization. Initially the MEMS devices had no specificity towards any semiochemicals, but after the functionalization they became selective towards the pheromone of the above mentioned insects. The functionalized MEMS devices act as a resonant mass sensor where they showed reduction in resonance frequency in the presence of the particular pheromone at room temperature. We covalently functionalized the SiO_2_ substrates by four different protocols so as to achieve one or more free amine functionality over each anchor site. The pheromones released by the class of female insects, *Helicoverpa armigera* and the like - primarily aldehydes such as *z*-11-hexadecenal and *z*-9-hexadecenal in a particular ratio - could then form an imine (schiff base) linkage with the available amine functional groups. For further studies with the pheromones and the functionalized MEMS devices, we took the mixture of *z*-11-hexadecenal and *z*-9-hexadecenal in a 25:1 weight ratio as this was the ratio found for the two aldehydes in the pheromone of *Helicoverpa armigera*^32^.

The first step of functionalization was the treatment of silane reagents with the piranha cleaned silicon dioxide (SiO_2_) surfaces. In order to occupy maximum number of functionalizable sites (-OH) available on the SiO_2_ surface, we treated the substrates with increasing concentrations of silane in toluene. The optimum concentration of the reagent was determined by the saturation of either the surface roughness value of the modified substrates as viewed using atomic force microscopy (AFM) (Fig. S2) or the decrease in the first order resonance frequency of the MEMS devices as recorded by laser doppler vibrometry (LDV) (Fig. S3). Both of the experiments, i.e. AFM and LDV, indicated that 4% of the aminosilane reagent (3-APTES) and 10% of the thiosilane reagent (3-MPTES) were optimum to occupy most of the functionalizable sites on the silicon dioxide substrates. The following steps were then carried out in each of the functionalization protocols for which the probable number of free amine groups increased theoretically from 1 in protocol 1 to 7 in protocol 2, 49 in protocol 3 and 343 in protocol 4.

**Figure 1:**
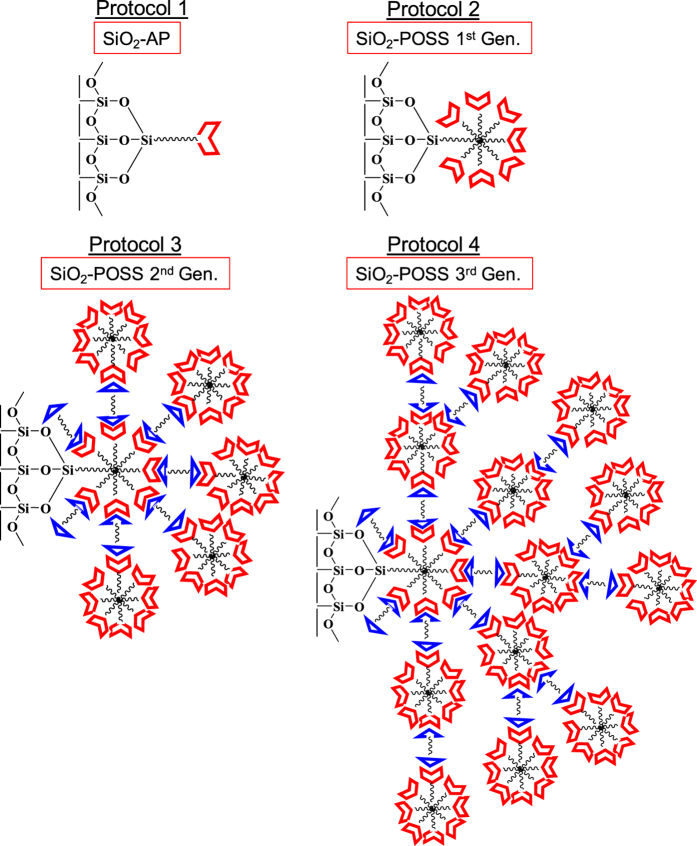
Schematic representation of the functionalized MEMS devices: Fabrication of the functionalized MEMS devices by four different protocols. The numbers of amine functionalities represented by the red curvature increase from protocol 1 to protocol 4 which also improves the relative affinity of the devices towards the pheromone.

In protocol 1, the piranha cleaned SiO_2_ substrates were reacted with 4% of 3-aminopropyl triethoxysilane (3-APTES) in toluene medium to generate at least one amine functionality (SiO_2_-AP) at each anchor site (**Scheme S1**). In protocol 2, the MEMS devices were first functionalized with 10% of 3-mercaptopropyl triethoxysilane (3-MPTES), then with the cross-linker, 3-(maleimido)-propionic acid N-hydroxysuccinimide ester, and dendrimeric moiety, amino functionalized polyhedral oligomeric silsesquioxane (POSS-NH_2_), in order to achieve at least seven free amino (-NH_2_) groups (SiO_2_-POSS 1st Gen.) at each anchor site (**Scheme S2**). In protocol 3, the functionalization steps were initially carried out similar to the protocol 2 and thereafter treated first with glutaraldehyde and then again with POSS-NH_2_ to generate a maximum of 49 free amino groups (SiO_2_-POSS 2nd Gen.) at each anchor site of the substrate (**Scheme S3**). In a similar fashion, the next generation of POSS was also designed over the SiO_2_ surfaces in protocol 4 to generate a maximum of 343 free amino groups (SiO_2_-POSS 3rd Gen.) at each anchor site (**Scheme S4**). Differential response towards the semiochemicals having carbonyl functionality was expected from these MEMS devices functionalized by four different protocols (Fig. 1) due to the presence of higher number of amino groups over the SiO_2_ surfaces functionalized by protocol 4 than by protocol 1.

### Characterization of the Functionalized MEMS Devices

**Figure 2:**
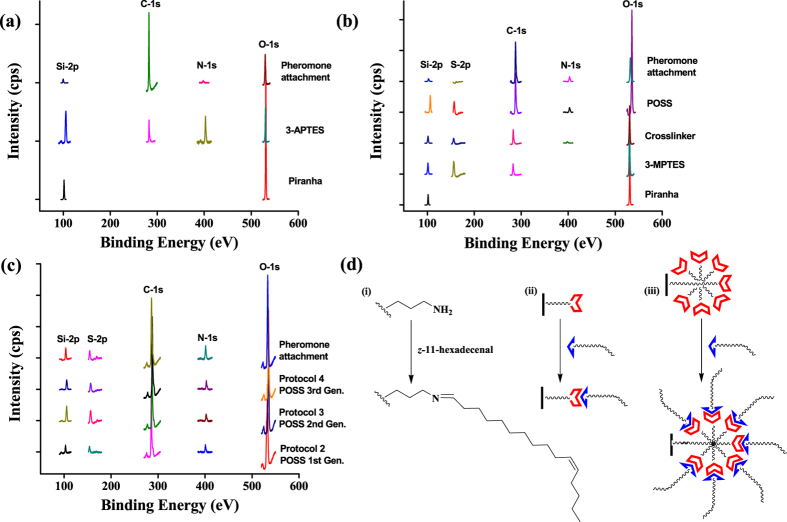
Characterization of the functionalized MEMS devices by XPS: XPS data of the silicon dioxide surfaces covalently functionalized with (**a**) protocol 1 and (**b**) protocol 2. The comparative XPS data among protocol 2, 3 and 4 is shown in (**c**). The XPS data for the pheromone attached with the functionalized SiO_2_ surface by protocol 3 is also represented in (**c**). The schematic representation of covalent attachement of pheromones with the aminated surfaces and a comparison of pheromone attachment between 3-APTES and POSS functionalized surfaces are shown in (**d**).

The successful covalent functionalization of the MEMS devices by the four different protocols was then characterized by various analytical techniques. X-ray photoelectron spectroscopy (XPS) showed the generation of C-1s peak at 283 eV and N-1s peak at 403 eV after the treatment of 3-APTES to the piranha cleaned SiO_2_ surface which ascertained the covalent attachment of amino silane moiety in protocol 1 (Fig. 2a)^33^. Again the presence of S-2p peak at 156 eV and C-1s peak at 282 eV confirmed the functionalization of 3-MPTES with the SiO_2_ surface in protocol 2, 3 and 4 (Fig. 2b). The concurrent generation of atomic peaks after each of the functionalization steps also corroborated the expectation and finally the large increase in C-1s intensity in each case after the exposure of female sex pheromones of *Helicoverpa armigera* proved the covalent bonding of the carbonaceous pheromones with the functionalized surfaces (Fig. 2c). The binding of z-11-hexadecenal with the aminated surface is also represented in Fig. 2d.

**Figure 3:**
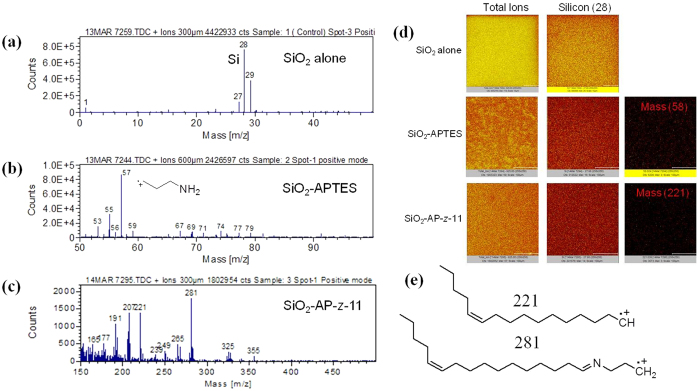
Characterization of the functionalized MEMS devices by ToF-SIMS: ToF-SIMS data of (**a**) piranha treated and (**b**) 3-APTES treated SiO_2_ surface. The spectral data for the z-11-hexadecenal exposed SiO_2_-APTES surface is shown in (**c**). The probable mass fragmentations which result in the mass peaks of 221 and 281 are shown in (**e**). The 2D images for the total ion counts and the average silicon ion density is depicted in (**d**) for the bare SiO_2_, SiO_2_-APTES and SiO_2_-AP-z-11 surfaces. The 2D images for the mass values corresponding to 58 and 221 are also shown in (**d**).

The functionalization of the SiO_2_ surfaces was further monitored by the time-of-flight secondary ion mass spectrometry (ToF-SIMS). The mass peak of 28 was observed in all the cases which indicated the presence of silicon atoms on the bare surfaces (Fig. 3a). The mass peak of 57 confirmed the attachment of aminopropyl unit to the SiO_2_ surface in protocol 1 (Fig. 3b) and the generation of mass peaks of 221 and 281 ascertained the presence of *z*-11-hexadecenal (z-11) units in the pheromone exposed surface of the substrates (Fig. 3c). The fragmented parts corresponding to these mass values are shown in the Fig. 3e. In 2D images, the total ion count and the silicon ion density increased as we moved from a bare SiO_2_ surface to the APTES functionalized SiO_2_ surface. But an insignificant change to the silicon ion density with continuous increase in total ion count was observed when we moved from SiO_2_-APTES surface to the SiO_2_-AP-*z*-11 surface, i.e., *z*-11-hexadecenal exposed SiO_2_-APTES surface (Fig. 3d). This observation is in direct correspondence with the functionalization steps of protocol 1 and confirms the successful covalent functionalization of SiO_2_ surfaces. Similarly the successful functionalization of SiO_2_ surfaces by protocol 2 was ascertained by the simultaneous presence of the fragmented mass peaks of 58, 74 and 880 values. The mass peaks of 58 and 74 confirmed the bonding of aminopropyl (part of POSS) and mercaptopropyl unit respectively to the SiO_2_ surface (Fig. S4a) and the presence of low intensity mass fragment of 880 proved the bonding of POSS unit to the SiO_2_ surface (Fig. S4b). The continuous increase in the total ion count and silicon ion density from protocol 1 to 4 confirmed the successful covalent functionalization of the SiO_2_ surfaces which is also reflected in their 2D images (Fig. S5).

### Stability of the MEMS Devices after Functionalization

After the successful characterization of the functionalized SiO_2_ surfaces by the four different protocols, the stability of the SiO_2_ based MEMS devices were monitored by scanning electron microscopy (SEM). It was found that the devices mostly sustained all the functionalization steps described in four protocols, but the cantilever with the maximum length of 86.6 μm could not withstand in protocol 3. It was also observed that most of the cantilevers, especially with the length of 86.6 and 36.5 μm, could not withstand the chemical treatments of protocol 4 (Fig. S6). Therefore, for further studies, we did not use the MEMS devices functionalized by protocol 4, although they would have the maximum number of amine functionalities.

### Reversibility of the Functionalized MEMS Devices

The change in the surface topography of the SiO_2_ surfaces after the functionalization by protocol 3 and after the exposure of pheromones was then examined using AFM and the increase in surface roughness was monitored (Fig. S7). The reversible use of these microstructures towards the pheromones could also be achieved by simple acid-base treatment. The devices were first dipped in glacial acetic acid (1 M solution, pH 2.4) at room temperature and then in dilute alkaline solution (1 M solution, pH 14.0). The acetic acid cleaved the Schiff base linkage between the aminated surface and the aldehyde group of the pheromone giving rise to protonated forms of the free pendant amine groups^34^. Subsequent dilute base treatment rendered the amine groups on silica surface neutral, which in turn made the amines available for further attachment with the pheromone molecules. This strategy to make the pheromone binding reversible to the substrates was confirmed from AFM studies where the surface roughness value regenerated to its original value after the acid-base treatment (Fig. S7d).

### Optical Sensing of Female Sex Pheromones of the Pests like *Helicoverpa armigera*

Next, the sensing of the female sex pheromones of the pests like *Helicoverpa armigera* was carried out with the functionalized cantilevers and fixed-fixed beams by protocol 1, 2 and 3. We employed the laser doppler vibrometry (LDV) to detect the attachment of pheromones with the functionalized microstructures. The cantilevers and fixed-fixed beams were specifically fabricated to be used as the pheromone mass sensor in a label free manner and in real time. These microdevices have received much attention among the researchers during the last few years for sensing of biomolecules^35^,^36^,^37^,^38^,^39^,^40^,^41^,^42^,^43^,^44^,^45^,^46^. The other advantages of these MEMS devices, that they are inexpensive and are readily available, have higher mass sensitivity and faster response time, make them ideal for large scale deployment. The sensing principle is based on the decrease in resonance frequency with the increase in pheromone mass over the surfaces of the devices. We observed a continuous decrease in the first resonant frequency with the increased exposure of the devices to the pheromone volatiles. The standard dynamic force field equation was used for both the cantilevers and fixed-fixed beams for the calculation of added mass over the functionalized surfaces where the relationship between the natural frequency of a MEMS device and the added mass is as follows:


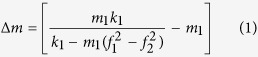


Here k_1_ is the spring constant of the MEMS device prior to functionalization, m_1_ is the mass of the functionalized device without any added mass of pheromones, f_1_ is the first resonant frequency prior to the exposure of the device to the pheromones and f_2_ is the resonant frequency with the added mass (Δm) of pheromones. It is to be noted that the value of Δk/k was negligibly small and hence the approximation made to calculate the increase in mass ignoring any changes in the stiffness is justified.

**Figure 4:**
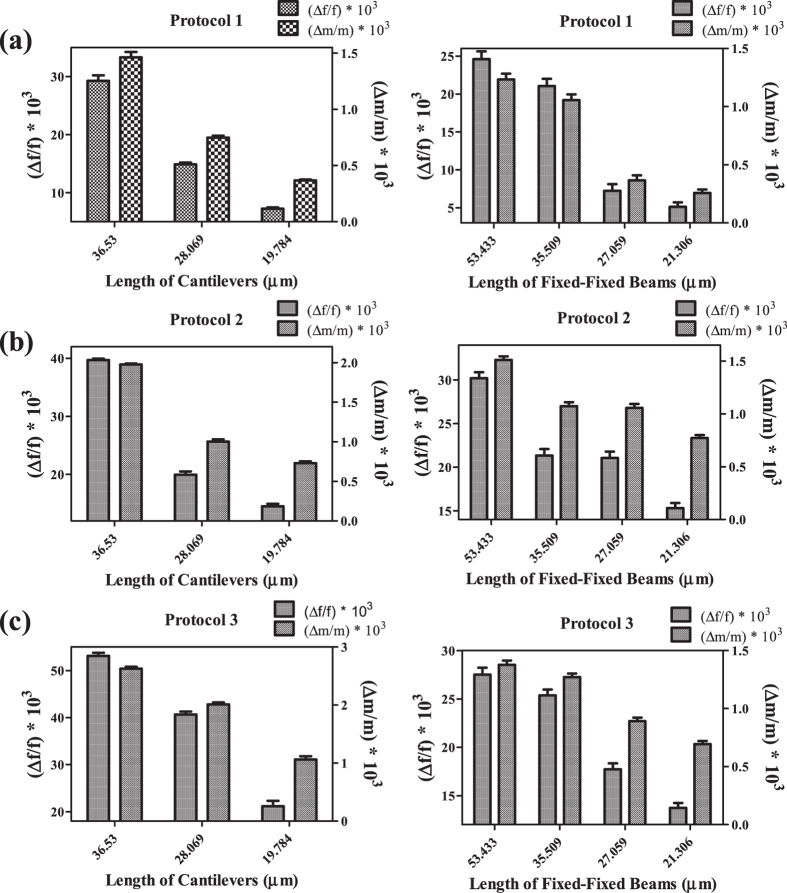
Comparing the sensing efficiency of the MEMS devices: Plot of Δf/f and Δm/m of the microfabricated cantilever (at left) and fixed-fixed beam (at right) arrays functionalized by (**a**) protocol 1, (**b**) protocol 2 and (**c**) protocol 3 after the exposure of the devices to ~5 ppm of pheromone concentration.

All of the MEMS devices functionalized by protocol 1, 2 and 3 showed a decrease in their first resonant frequency after the exposure to the same concentration of pheromones, but we found that the response of the cantilevers was higher when compared to that of the fixed-fixed beams functionalized by the same technique (Fig. 4). Again the change in the first resonance frequency (i.e., Δf/f) was observed to be much superior for the cantilevers functionalized by protocol 3 than the other two protocols (Fig. S8). This trend was same for the increase in mass, i.e. Δm/m, over the MEMS devices (Fig. 4) and the effective number of pheromone molecules attached to the MEMS devices (Fig. S8). It could be said that the presence of higher number of amine functionalities per anchor site of the devices functionalized by protocol 3 might be the reason for its superior sensitivity over other protocols.

It was also observed that the relative sensitivity of the functionalized devices improved with the increase in length of the cantilevers, which could be explained from the increased flexibility of the devices to show larger reduction in the first resonant frequency with the greater increase in added mass of pheromones (Fig. 4). Hence the cantilever functionalized by protocol 3 and having the length of 36.53 μm was the most sensitive MEMS device among all in sensing of the female sex pheromones of *Helicoverpa armigera*. The plot of Δf/f vs. Δm/m show good agreement with a linear fit which shows excellent applicability of these cantilever sensors towards this application (Fig. S9a). The limit of detection was found to be 4.06 ± 0.5 fg of attached pheromone mass over the cantilever (length 36.53 μm) surface functionalized by protocol 3, which was well below the level of pheromones found in an agricultural field or vineyard during a pest infestation as per the OECD Monograph Guidance – Pheromones and Semiochemicals - September 2002^31^ (Fig. S9b). Hence these cantilevers can be deployed in an agricultural field to determine the presence of pheromones much before the occurrence of large scale pest infestation. This strategy will also help farmers to schedule appropriate pest control measures in a confined region with alert of impending pest attack instead of applying the pesticides all over the field. The limit of quantification for this cantilever was found to be 12.3 ± 0.9 fg of the attached pheromone mass over the functionalized surface. Interestingly the reversibility of the cantilevers was also achieved by a simple acid-base treatment as was explained in the previous section.

### Selectivity of the Functionalized MEMS Devices when compared with various Interfering Semiochemicals

Further the selectivity of the particular cantilever functionalized by protocol 3 was determined in the presence of various interfering semiochemicals commonly found in an agricultural field infested by *Helicoverpa armigera* and like. It is observed that some of the common interferences for the detection of female sex pheromone of *Helicoverpa armigera*, those are normally present in an agricultural field, are the pheromones of grass grub beetle, white grub beetle, red ant, leaf cutting ant, house cricket and the allomones of eastern grasshopper, brown rat etc. Some of the other common interfering semiochemicals are the attractants of fruit fly, microworm and the kairomone of corn earworm etc. The active functional group of these interfering agents also varies from species to species^47^,^48^,^49^,^50^,^51^,^52^,^53^,^54^,^55^,^56^ and these are tabulated below. The results obtained from the functionalized cantilever when exposed to these volatile interfering semiochemicals are also represented in Table 1. Interestingly we found that almost no change in the first order resonant frequency of the functionalized cantilever and thus these chemicals served as negative controls (Fig. S10).

But significant decrease in resonant frequency was found only in the presence of pheromones having carbonyl functionalities, especially free aldehyde groups. It was found that the functionalized cantilever is very selective towards the female sex pheromone of *Helicoverpa armigera, Scirphophaga incertulas* and like (Fig. S10). Again these cantilevers when exposed to the same concentration of pheromones, the decrease in resonance frequency was observed to be more in case of *Scirpophaga incertulas* than for *Helicoverpa armigera*. The plausible reason for this might lie in the difference in ratio of the two major components of their pheromones, *z*-11-hexadecenal and *z-*9-hexadecenal as 25:1 for *Helicoverpa armigera*^32^ to 3:1 for *Scirpophaga incertulas*^57^. Thus the functionalized cantilever is compared for its specificity towards the female sex pheromone of certain agriculturally important pests like *Helicoverpa armigera*.

### Experiment with Live Insects at Real Field Conditions

**Figure 5:**
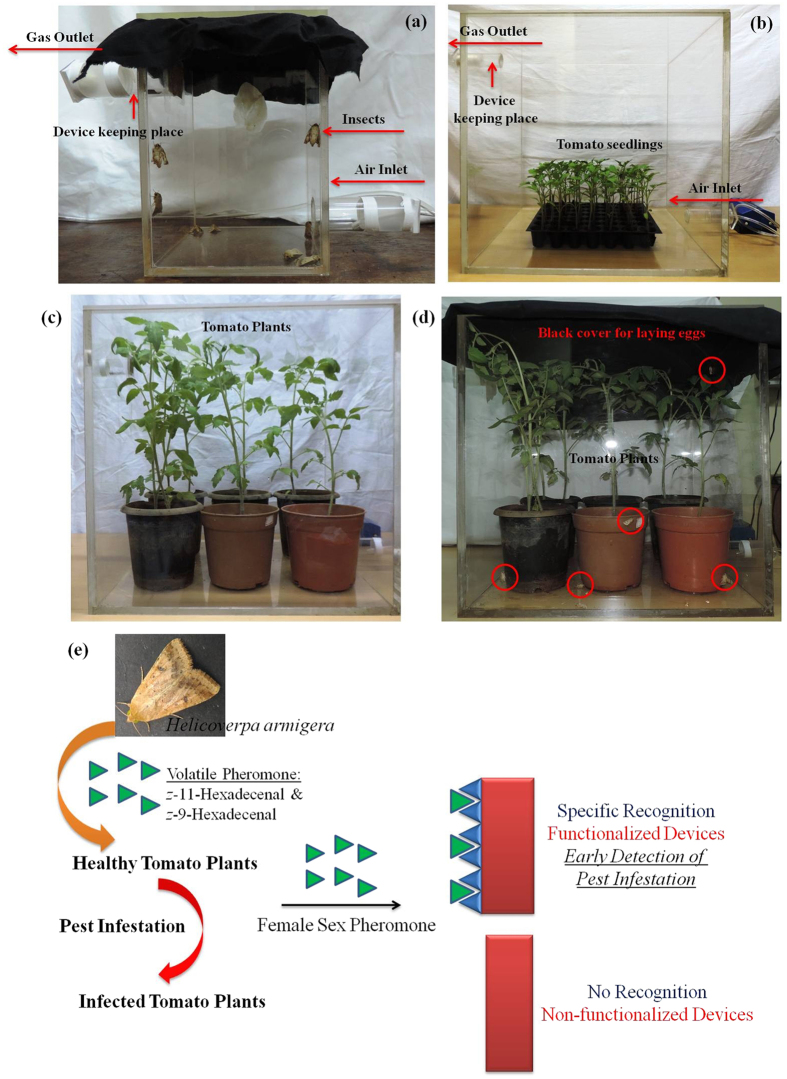
Data in presence of live insects at field prototype conditions: (**a**) Sensing of pheromones by the functionalized devices in a box with eight of the female insects. Selectivity of the devices from the kairomones of (**b**) tomato seedlings and (**c**) tomato plants is represented. A mimic of the field condition with six of the tomato plants and four of each male and female insect is shown in (**d**). Schematic representation for the detection of female sex pheromone of *Helicoverpa armigera, Scirphophaga incertulas* and the like pests, prior infestation, by the use of covalently functionalized MEMS devices is demonstrated in (**e**).

We have also tested the efficiency of these functionalized devices with the pheromones released by the live *Helicoverpa armigera* pests. The unmated female insects were separated in pupa stage from the male ones, cultured separately and initially eight of the female insects were kept in a box covered with a black cloth on top. A continuous flow rate of air was maintained from the bottom of the box to direct the pest excreted semiochemicals to the top channel of the box, where the functionalized devices were placed (Fig. 5a). The devices were taken out after a finite time interval and investigated for their change in the first resonant frequency. Significant decrease in resonant frequency was found at each time interval of 6, 12 and 24 h (Fig. S11). We also monitored the change in resonant frequency of the devices in presence of various kairomones released by tomato seedlings and plants (Fig. 5b,c) during the stipulated time intervals, but we encountered almost no change in their frequency which further demonstrated the selectivity of the functionalized devices towards the pheromones of *Helicoverpa armigera*. The actual field condition was simulated in a large box containing six tomato plants and four of each male and female *Helicoverpa armigera* insects. The top of the box was covered by a black cloth where the female pests could lay eggs (Fig. 5d). Again the devices showed significant decrease in resonant frequency establishing the functionalized devices as a unique pheromone sensor for *Helicoverpa armigera*.

## Conclusions

Herein we have developed a MEMS based pheromone sensor specific for the female sex pheromone of lepidopterous pest such as *Helicoverpa armigera* of cotton, tomato, rice, pigeonpea and chickpea etc. We have fabricated and successfully functionalized the silicon dioxide based MEMS devices in order to receive the response in terms of change in resonant frequency when the device is exposed to the volatile pheromones. The novel functionalization protocol makes these devices selective and sensitive towards the pheromone. The durability of the functionalization for prolonged testing period is guaranteed due to the covalent bonding. The chemical functionalizations of the devices have photochemical and thermal stability which makes them environment friendly. The functionalized devices can be used at ambient temperature and at natural atmospheric conditions, even in rainy season or in peak summer under bright sunshine. The maintenance cost for these devices is practically zero and the functionalized devices are also reusable due to their engineered reversibility. The trouble-free transportation of these pheromone sensors aids to the commercial applicability of this approach in order to successfully carryout the translation from the research laboratory to the agricultural field. Moreover any resonant mechanical structure such as beams, plates or membranes can be modified to immobilize the pheromones using these unique surface functionalization protocols described here and detect their selectively from various interfering substances. Further the limit of detection found for these devices is much lower than the level of pheromone concentration found in an agricultural field or vineyard during pest infestation. The devices may also quantitatively estimate the concentration of pheromones present in an agricultural field. The mass scale sensor development can now be carried out for these functionalized devices for direct field deployment in order to detect early pest attack. The farmers can then take necessary actions in a confined region of alerted pest attack without applying the pesticides all over the field. All of these features heighten the potential of these covalently functionalized devices for commercial use.

## Methods

### Fabrication of Cantilever and Fixed-fixed Beam Arrays

Cantilever is a projecting substrate structure which is supported at one end only and fixed-fixed beams are the ones those are supported from both the ends. The fabrication protocol for these microcantilever and microbeam arrays is as follows:

[1] Cleaning and Growth of Silicon Dioxide Layer:

A silicon wafer of 500–550 μm thickness (type P, dopant boron, orientation <100>, 100 mm diameter and, resistivity 0–100 was taken and cleaned well with 20 mL of piranha solution (H_2_SO_4_:H_2_O_2_ = 9:1) for 5 min. It was washed repeatedly with distilled water to remove metallic and organic contaminants from the surface and then a 1 μm layer of silicon dioxide was thermally grown over it by nano pyrogenic furnace.

[2] Photolithography:

- Dehydration and Spin Coat: A properly cleaned wafer was first dehydrated at 250 °C for 10 min by keeping it over a hot plate to evaporate all of the surface moisture. Then it was spin coated with AZ5214E, photoresist (PR), of nearly 1.5 μm thickness at 6000 rpm for 40 seconds and baked to about 110 °C for 2 min to evaporate the solvents in PR.
- Alignment for pattern transfer: A 3 inch DRIE lithomask was fixed over the treated wafer and kept under UV exposure for 2 sec in EVG 620 double sided mask aligner (50 mill joules/cm2). The mask was developed by dipping it in the solution of AZ351B:H_2_O in a ratio of 1:4 for 30 sec. It was washed with distilled water, dried under nitrogen and finally heated at 110 °C for 4 min to get the pattern for the expected microstructures. The developed cantilevers and fixed-fixed beams were observed under the optical microscope.

[3] Dry Etch to release the pattern:

First formalin oil was put on a large carrier wafer to prepare sticky base for the sample. The sample was loaded inside the reactive ion etching chlorine (RIE-Cl) chamber and followed by the three steps.

- Anisotropic plasma etch of silicon: First silicon dioxide (SiO_2_) was plasma etched with 5 torr of pressure and 50 power for 6 min.
- Isotropic Si etch: Secondly silicon was etched with 7.5 torr of pressure and 30 power for 3 min and
- Oxygen etch: Lastly photoresist was removed by oxygen etching with 0 torr pressure and 150 power for 4 min.

The completely released devices were carefully diced from the wafer and proceeded for the functionalization steps. Four different cantilevers and fixed-fixed beams each of which distinguishable from the other based upon its length were fabricated. The cantilevers had the lengths of 86.6, 36.5, 28.1 and 19.8 μm respectively and the fixed-fixed beams had the lengths of 53.4, 35.5, 27.1 and 21.3 μm respectively. The cantilevers had the uniform width of 5.08 μm, whereas the fixed-fixed beams had uniform width of 4.74 μm. Each of the structure had a uniform thickness of 1.04 μm.

### Covalent Surface Functionalization Protocols

The obtained silicon dioxide based microstructures were covalently functionalized by four different protocols. We had chosen B-doped silicon dioxide surface^58^ having free hydroxyl groups which can be covalently modified by silane chemistry easily. Also during the thermal growth of silicon dioxide and followed by chemical modification of SiO_2_ layer, we have chosen <111> surface of silicon due to its surface atomic arrangement which leads to densely packed layers and low density of dangling bonds^59^.

At first we have cleaned the surfaces of the silicon dioxide based substrates by dipping in 20 mL of piranha solution (H_2_SO_4_:H_2_O_2_ = 9:1) for 5 min to remove the organic and metallic contaminants. The temperature of the solution is maintained at 85 °C and H_2_O_2_ was added carefully to replenish the temperature. These cleaned devices were undertaken for the further functionalization steps to create at least one anchor site with one or multiple amine groups.

Protocol 1:

Step 1: The hydroxyl groups on the cleaned surface of substrates were functionalized with 4% of 3-aminopropyl triethoxy silane (3-APTES) in 10 mL of toluene under N_2_ atmosphere at room temperature for 2 h. After the reaction time is over, the surfaces were rinsed with distilled water repeatedly and finally dried under nitrogen flow.

Protocol 2:

Step 1: The hydroxyl groups on the cleaned surface of substrates were functionalized with 10% of 3-mercaptopropyl triethoxy silane (3-MPTES) in 10 mL of toluene under N_2_ atmosphere at room temperature for 4 h. Then the surfaces were rinsed with fresh toluene repeatedly and finally dried under nitrogen flow. The surfaces were reacted immediately as described in the next step or it was kept under xylene to protect the surfaces from aerial oxidation.

Step 2: The free thiol groups on the surfaces were reacted with a crosslinker, 3-(maleimido)-propionic acid N-hydroxysuccinimide ester in 10 mL of dimethylformamide (DMF) for 5 h and then rinsed properly with water and ethanol. Again the surfaces were dried under nitrogen flow before proceeding for the next step.

Step 3: The surface immobilized N-hydroxysuccinimido group was further reacted with an amino functionalized polyhedral oligomeric silsesquioxane (POSS-NH_2_)^60^ moiety in 12 mL of DMF:H_2_O = 3:1 mixture. Ten equivalents of triethylmine (Et_3_N) and 0.1 equivalent of dimethylaminopyridine (DMAP) with respect to POSS-NH_2_ were also added to the same mixture and kept for overnight. It was rinsed properly with distilled water repeatedly and finally dried under nitrogen flow.

Protocol 3:

Step 1–3: These steps were similar as described in protocol 2.

Step 4: The aminated surface was then reacted with 10% of glutaraldehyde in 10 mL of PBS buffer (pH 7.4) for 4 h at room temperature. Then it was washed with distilled water and dried under nitrogen flow.

Step 5: This step is same as with the step 3 of protocol 2.

Protocol 4:

Step 1–5: These steps were similar as described in protocol 3.

Step 6–7: These steps are same as with the step 4 and 5 of protocol 3 respectively.

### Optical Microscopy

The length and width of the MEMS devices were examined under Leica DIC optical microscope.

### Scanning Electron Microscopy (SEM)

The successfully released MEMS devices and the stability of them after all of the covalent functionalizations were monitored by Ultra 55, Field Emission Scanning Electron Microscope (FESEM) instruments from Carl Zeiss.

### Atomic Force Microscopy (AFM)

AFM images were obtained by JPK instruments using NanoWizard JPK00901 software in tapping mode. Analyses of the AFM images were processed using JPK data analyzer software. The cleaned surfaces were glued over a plate using a very small piece of double-sided tape and the images were recorded using silicon AFM tip with a resonance frequency about 300 kHz and force constant of 40 N/m.

### X-ray Photoelectron Spectroscopy (XPS)

In order to determine the surface abundance of atoms, X-ray photoelectron spectroscopy (XPS) was performed using a Kratos Axis ULTRA spectrometer (Shimadzu) outfitted with a non-monochromatic Al Kα X-ray source (105 W). Samples were electrically grounded for XPS measurements, and the binding energy scale was referenced to the Fermi level. Analyzer pass energy for wide (survey) sccm was 160 eV and for high resolution was 20 eV. The accelerating voltage was 105 kV and the current was 10 mA. Lense mode was kept in hybrid and SAC and STC vacuum level was kept at 1.8 e^−8^ and 1 e^−7^ torr respectively during the data recording. Samples were ultra-cleaned prior recording of the XPS data to remove the surface availability of unwanted atoms.

### Time of Flight-Secondary Ion Mass Spectrometry (ToF-SIMS)

To determine the nature of atomic and molecular species from the functionalized solid surface, we used time-of-flight secondary ion mass spectrometry (ToF-SIMS)^61^. This was performed using PHI TRIFT V nanoTOF model manufactured by Physical Electronics, USA. The data were acquired in the static mode using single beam in surface analytical regime where the samples were kept in UHV mode for surface acquisitions and sample chamber vacuum was typically 6.2e^−7^ Pascal region of pressure. Acquisition was done by a focussed 30 KV Ga ion source in the LMIG gun which was rastered in 300 × 300 μm area to induce the desorption and ionization of atomic and molecular species from the functionalized surfaces with the beam current typically around 7 nA. The resulting secondary ions were accelerated into the mass spectrometer where they were mass separated by measuring the time-of-flight from the sample to the detector and a mass spectrum was recorded. Surface spectra were acquired in positive ion mode with a mass range of 0–1500 amu on 3–5 sections, for 5 min each, on two samples per set. A 2D Image was generated by rastering a finely focussed ion beam across the sample surface.

### Laser Doppler Vibrometry (LDV)

Advanced 3-D dynamic response data were collected from the functionalized MEMS devices by MSA-500 (Polytech) instruments before and after the attachment of the pheromones. The resonant frequency due to the base vibration was measured by the laser doppler vibrometer (LDV) to quantify the change in displacement and velocity of the vibrating structures without making any surface contact. The measured resonant frequency was extracted by analyzing the vibration spectrum with the use of polytech acquisition software.

### Rearing of *Helicoverpa armigera*

Rearing of *Helicoverpa armigera* on artificial, semi-synthetic diet was carried out according to the well established protocol. The composition of the diet was as follows: Fraction A: kabuli gram (105 g), methyl para-hydroxyl benzoate (2 g), sorbic acid (1 g), yeast tablets (2 capsules of 500 mg each). Fraction B: agar agar (12.75 g) and Fraction C: ascorbic acid (3.25 g), multivitamin (2 capsules of 400 mg each), vitamin E (2 capsules of 268 mg each), streptomycin sulphate (0.25 g).

The room temperature was maintained at 26 ± 1 °C and relative humidity was controlled at ~60–70%. Then the ingredients of fraction A were mixed in 390 mL of water and blended for 2 min. Fraction B was boiled in 390 mL of water and added to fraction A in the blender and the resulting mixture was blended for a minute. The contents of fraction C were then added to this and blended again for a minute. The diet was then dispended into sterilized glass vials or plastic container to a height of 2.5 cm and plugged with cotton wool or pin holed lid. The larvae were transferred at the rate of single vial or container and the pupae formed were collected after 20–25 days. The compartments in the inner multicellular tray divided the diet in the basal tray into pieces. The larvae were then individually placed in each of the compartments through the hole. The lid lined with wire mesh was used for covering the holes of the inner tray. The larval period lasted for 20–25 days, pupal period for 7–10 days, and adult longevity of 7–10 days.

### Cultivating tomato plants

Tomato (*S. lycopersicum*) var., Arka Saurabh seeds were purchased from the Indian Institute of Horticultural Research (IIHR), Bangalore. A commercial peat-based substrate of biopeat coconut pith compost and agrosil (a mixture of phosphates that stimulates rooting) was used. The seeds were sown in trays (52 cm × 27 cm) and placed in a cultivation chamber at 25 °C. Later, the seedlings were transplanted into pots. On fully matured tomato plants (after 60 days), the larvae of *Helicoverpa armigera* were innoculated.

## Additional Information

**How to cite this article**: Moitra, P. *et al*. A novel bio-engineering approach to generate an eminent surface-functionalized template for selective detection of female sex pheromone of *Helicoverpa armigera. Sci. Rep.* **6**, 37355; doi: 10.1038/srep37355 (2016).

**Publisher’s note:** Springer Nature remains neutral with regard to jurisdictional claims in published maps and institutional affiliations.

## Supplementary Material

Supplementary Information

## Figures and Tables

**Table 1:** List of common interfering semiochemicals released from various species and their interactions with the functionalized cantilever for the detection of female sex pheromone of certain hazardous pests.

Job Number	Name of the Species	Name of One of the Semiochemical Present in the Mentioned Species	Results Obtained with the Functionalized Cantilever
1	*Costelytra zealandica* (White), the Grass grub beetle^47^	phenol (pheromone)	Insignificant change
2	*Dasylepida ishigakiensis* (Niijima & Kinoshita), the White grub beetle^48^	propan-2-ol (pheromone)	Insignificant change
3	*Myrmica rubra* (Linnaeus), the Red ant^49^	ethanol (pheromone)	Insignificant change
4	*Acromyrmex octospinosus* (Reich), the Leaf cutting ant^50^	furfuryl alcohol (pheromone)	Insignificant change
5	*Acheta domesticus* (Linnaeus), the House cricket^51^	propanoic acid (pheromone)	Insignificant change
6	*Romalea microptera* (Palisot de Beauvois), the Eastern lubber grasshopper^52^	catechol (allomone)	Insignificant change
7	*Rattus norvegicus* (Berkenhout), the Brown rat^53^	diethyl ether (allomone)	Insignificant change
8	*Anastrepha ludens* (Löw), the Mexican fruit fly^54^	ammonia (attractant)	Insignificant change
9	*Panagrellus redivivus* (Linnaeus), the Microworm^55^	ethyl propionate (attractant)	Insignificant change
10	*Helicoverpa zea* (Boddie), the Corn earworm^56^	ethylene (kairomone)	Insignificant change
11	**Helicoverpa armigera* (Hubner), the Cotton bollworm^32^*	*z*-11-hexadecenal: *z-*9-hexadecenal = 25:1	Significant change
12	*Scirpophaga incertulas* (Walker), the Yellow rice stemborer^57^	*z*-11-hexadecenal: *z-*9-hexadecenal = 3:1	More significant change
