# Supplementary material for: A novel bio-engineering approach to generate an eminent surface-functionalized template for selective detection of female sex pheromone of Helicoverpa armigera
Source: Sci Rep. 2016 Nov 28;6:37355. doi: 10.1038/srep37355 (PMC5124941; doi:10.1038/srep37355)
Supplement: Supplementary Information [file srep37355-s1.pdf]

Supporting Information

A novel bio-engineering approach to generate an eminent surface-functionalized template for selective detection of female sex pheromone of *Helicoverpa armigera*

Parikshit Moitra[1,†], Deepa Bhagat[2], Rudra Pratap[3] and Santanu Bhattacharya*[,1,†]

[1]Department of Organic Chemistry, Indian Institute of Science, Bangalore 560 012 (India).

[2]National Bureau of Agriculturally Insect Resources, P.B. No. 2491, H. A. Farm Post, Bangalore 560 024 (India).

[3]Centre of Nano Science and Engineering, Indian Institute of Science, Bangalore 560 012 (India).

[†]Present Address: Director's Research Unit, Indian Association for the Cultivation of Science, Jadavpur, Kolkata 700032 (India).

*Corresponding author. Email: sb@orgchem.iisc.ernet.in, Phone: (91)-80-2293-2664, Fax: (91)-80-2360-0529

Contents **Page number**

Schemes S2

Figures S5

Schemes.

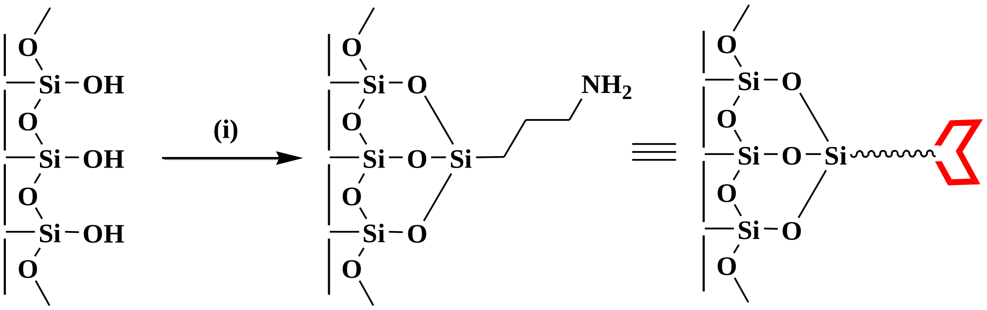

Scheme S1. Surface functionalization steps with protocol 1 in order to achieve at least one amine functionality per anchor site: The treatment with (i) 4% of 3-aminopropyl triethoxysilane (3-APTES) solution in toluene for 2 h at rt.

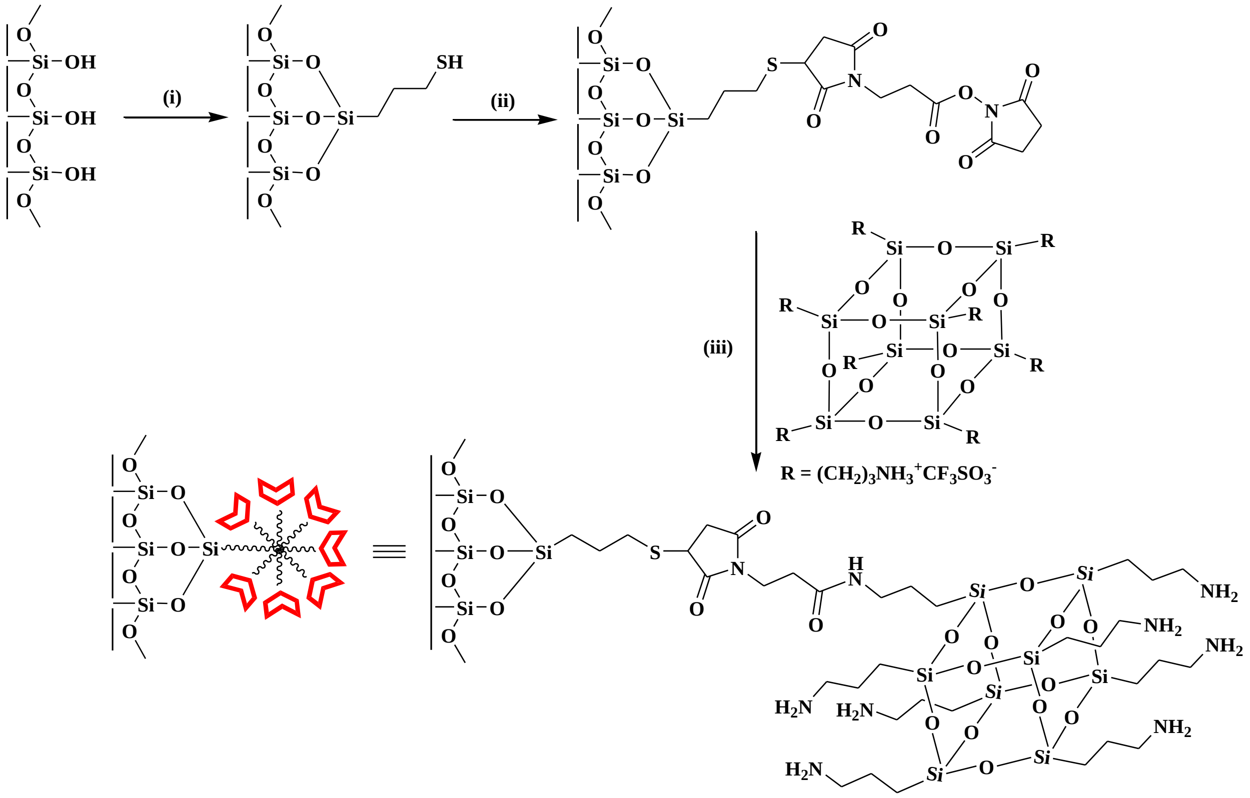

Scheme S2. Surface functionalization steps employed with the protocol 2 in order to achieve at least one anchor site with seven amine functionalities: The treatments with (i) 10% of 3-mercaptopropyl triethoxysilane (3-MPTES) solution in acetone for 2 h at rt; (ii) 3.76 mM

solution of the cross-linker, 3-(maleimido)propionic acid N-hydroxysuccinimide ester in DMF and left for overnight with constant agitation at rt; (iii) polyhedral oligomeric silsesquioxane (POSS) (8 mg/mL) in DMF/H2O (3:1) with 10 equivalents of Et3N and allowed to react overnight at rt.

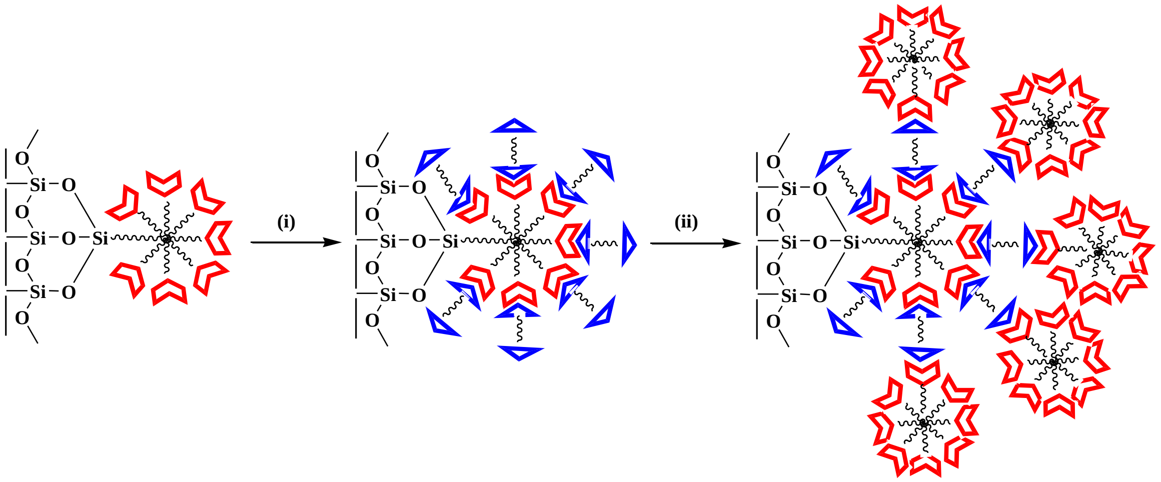

Scheme S3. Schematic representation of the surface functionalization steps with the protocol 3 in order to achieve a maximum of 49 amine functional groups at each of anchor site: The treatments with (i) 5% of glutaraldehyde solution in PBS buffer (pH 7.4) for 2 h at rt; and (ii) polyhedral oligomeric silsesquioxane (POSS) (8 mg/mL) in DMF/H2O (3:1) with 10 equivalents of Et3N and allowed to react overnight at rt.

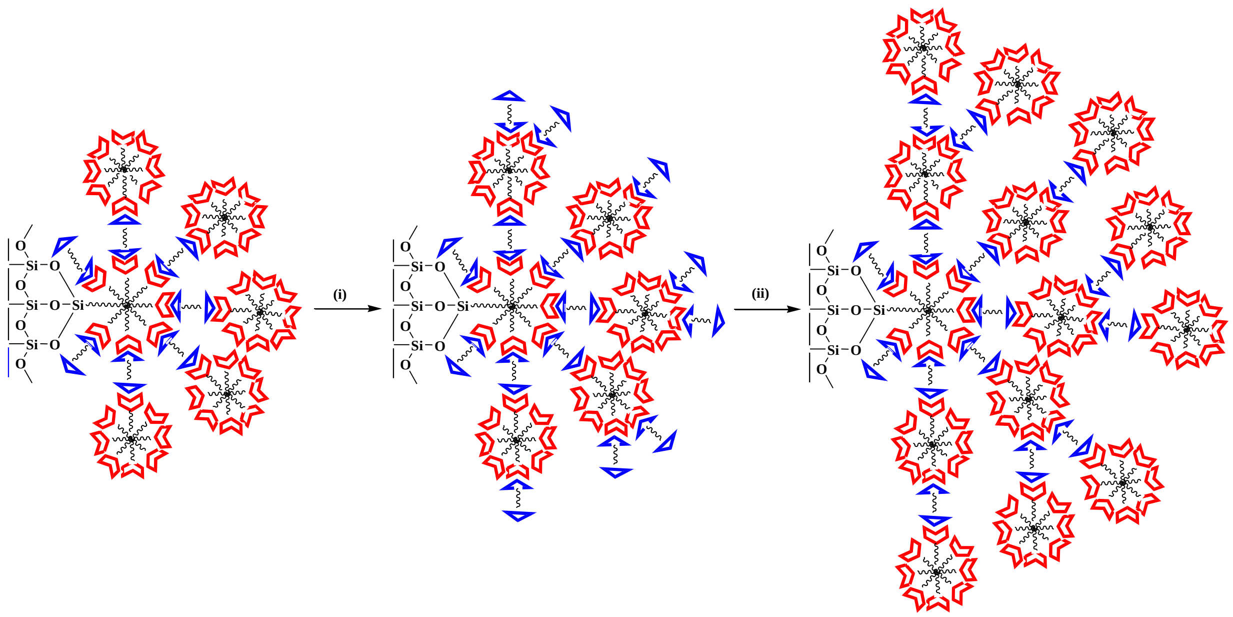

Scheme S4. Schematic representation of the surface functionalization steps with the protocol 4 in order to achieve a maximum of 343 amine functional groups at each of the anchor sites: The treatments with (i) 5% of glutaraldehyde solution in PBS buffer (pH 7.4) for 2 h at rt; and (ii) polyhedral oligomeric silsesquioxane (POSS) (8 mg/mL) in DMF/H2O (3:1) with 10 equivalents of Et3N and allowed to react overnight at rt.

Figures.

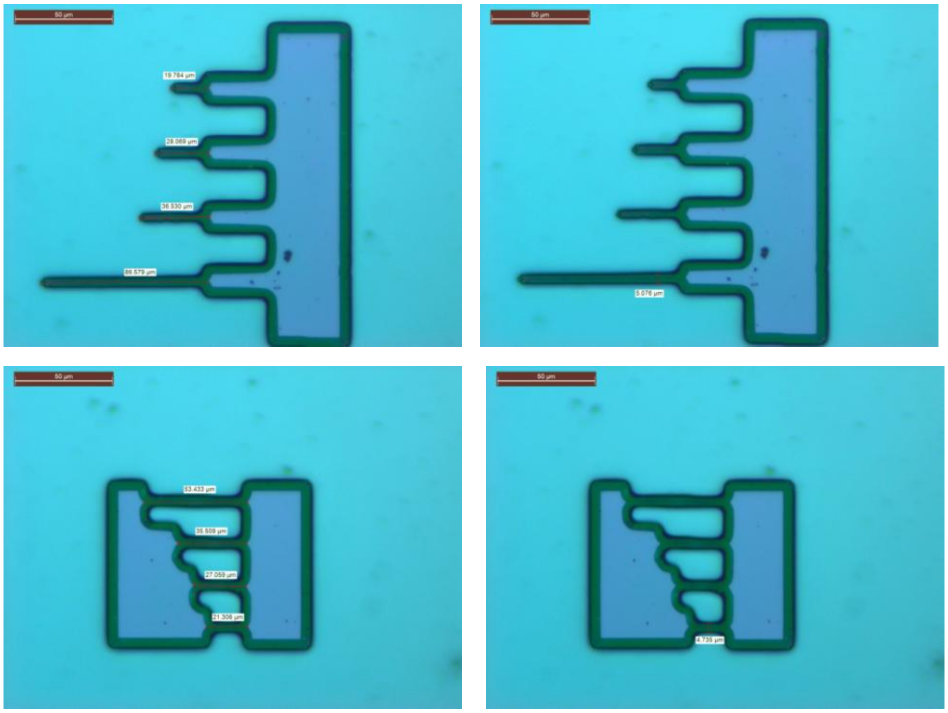

Figure S1. Calculation of length and width of the MEMS devices: Optical micrographs of the fabricated silicon dioxide based microcantilevers (top) and microbeams (bottom). The devices had uniform width, but were distinguishable from each other based upon their lengths.

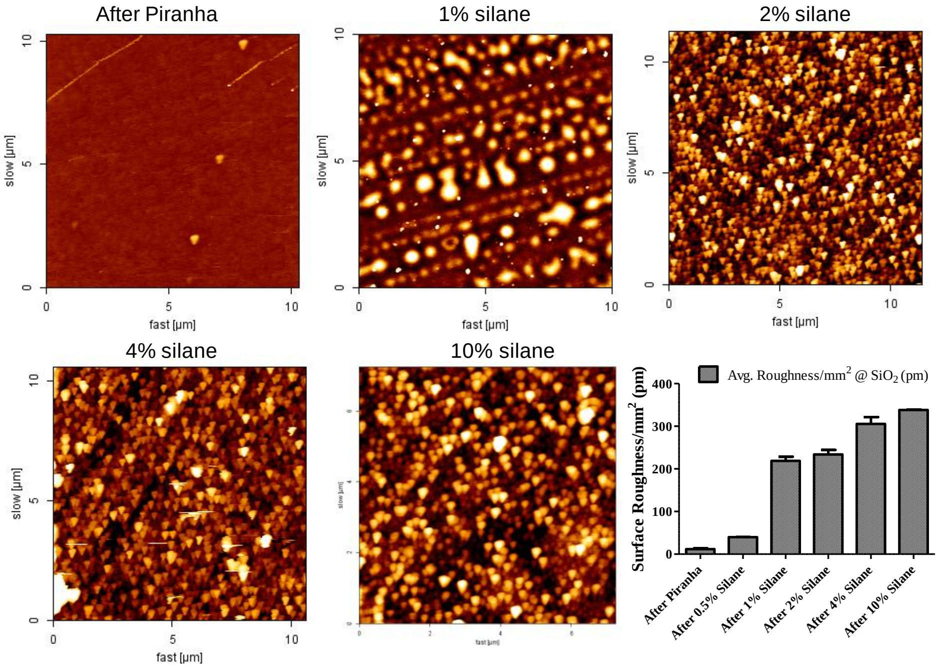

Figure S2. Determination of % silane concentration from AFM: AFM images of the silicon dioxide surfaces after the treatment with piranha, 1%, 2%, 4% and 10% of silane reagents in toluene. Histogram depicts the change in surface roughness after each of the increasing concentrations of silane reagents.

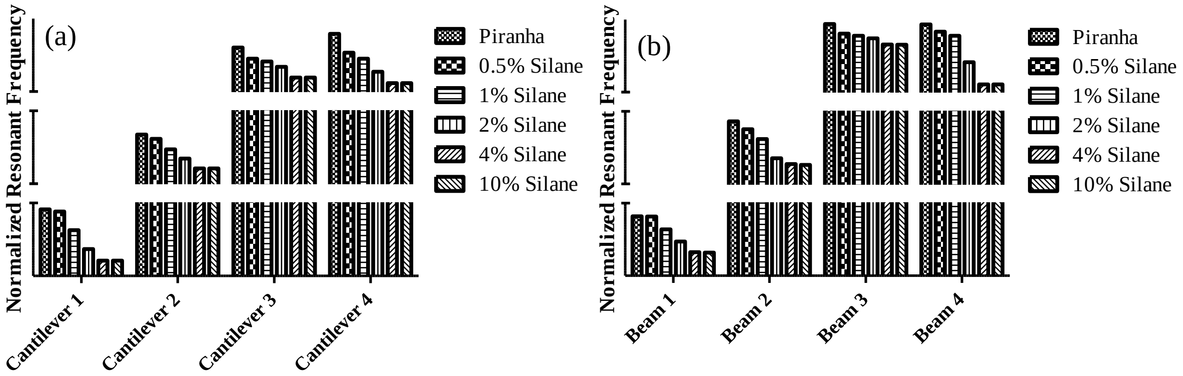

Figure S3. Determination of % silane concentration from LDV: Histograms depicting the change in the first order resonant frequency of the cantilevers (at the left) and the fixed-fixed beams (at the right) with increasing concentration of silane in toluene.

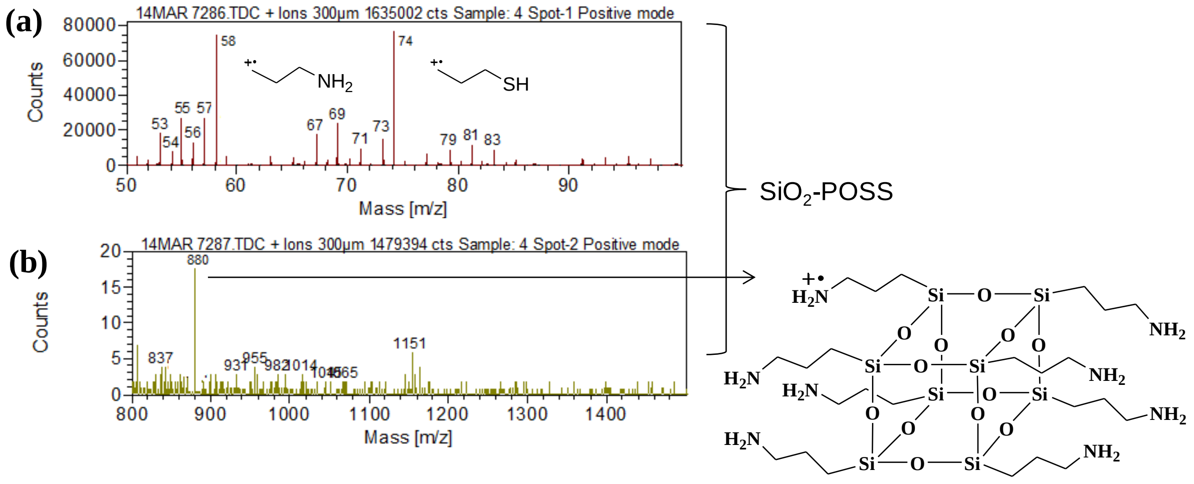

Figure S4. Characterization of the covalent functionalization by protocol 2: ToF-SIMS data of the silicon dioxide surfaces functionalized using the protocol 2.

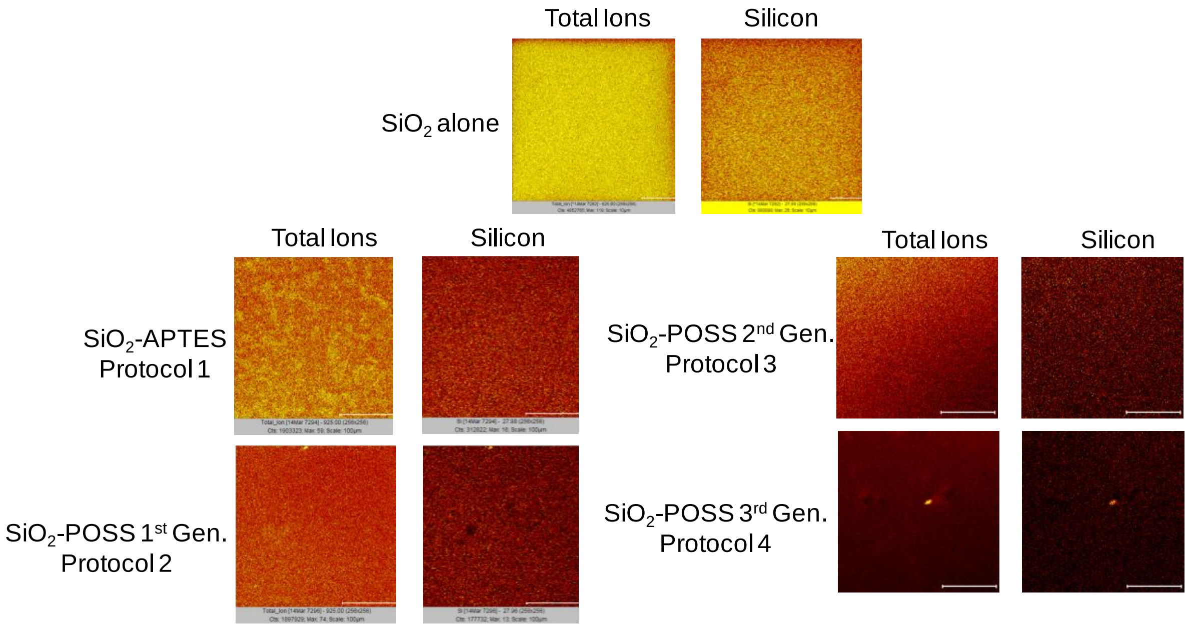

Figure S5. ToF-SIMS 2D images for the total ion counts and the average silicon ion density: Comparison of the bare silicon dioxide surface with the surfaces covalently functionalized using the protocol 1, 2, 3 and 4 respectively.

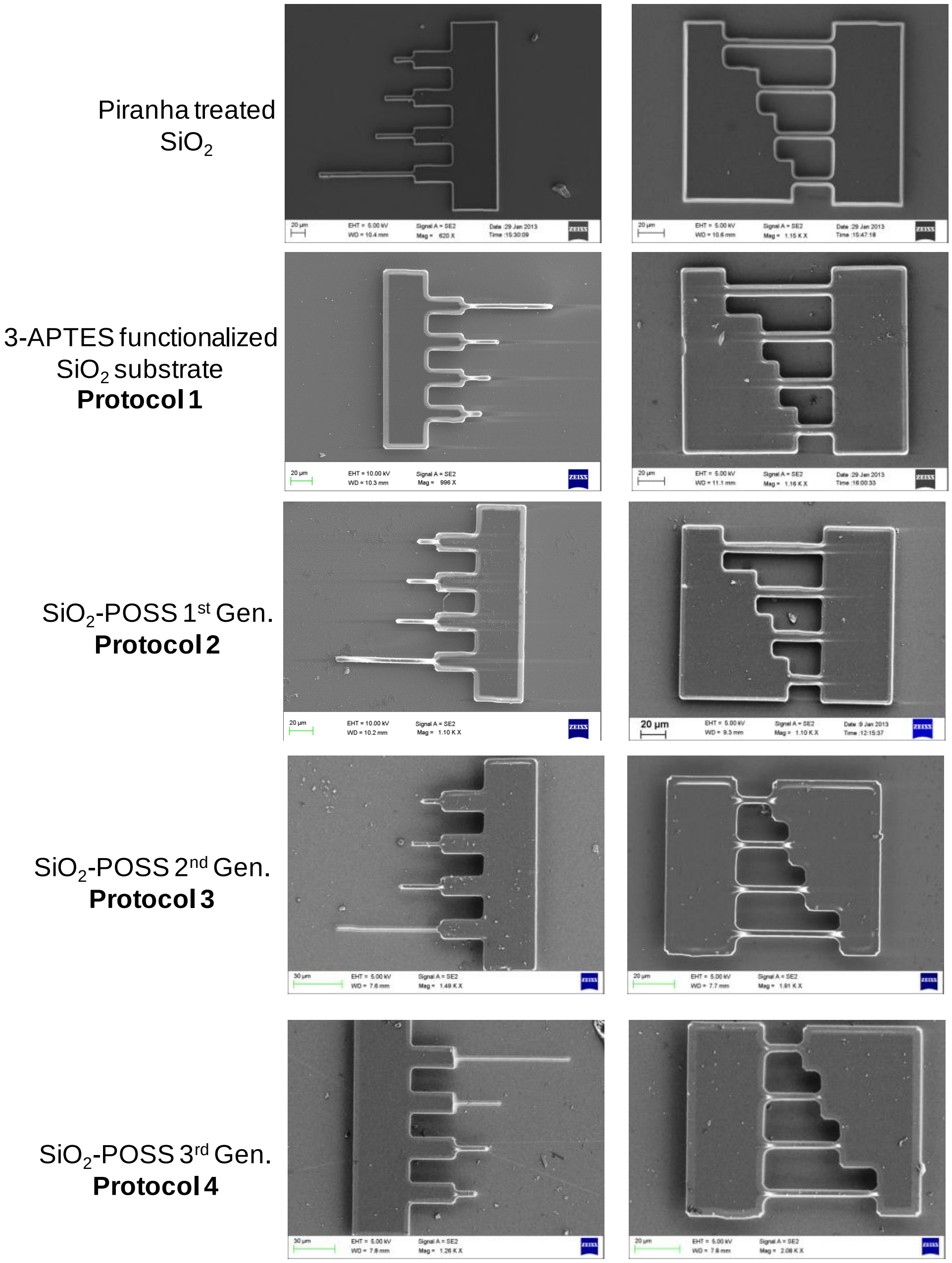

Figure S6. Stability of the functionalized MEMS devices: SEM images of the cantilevers (at the left column) and fixed-fixed beams (at the right column) after each of the functionalization protocols.

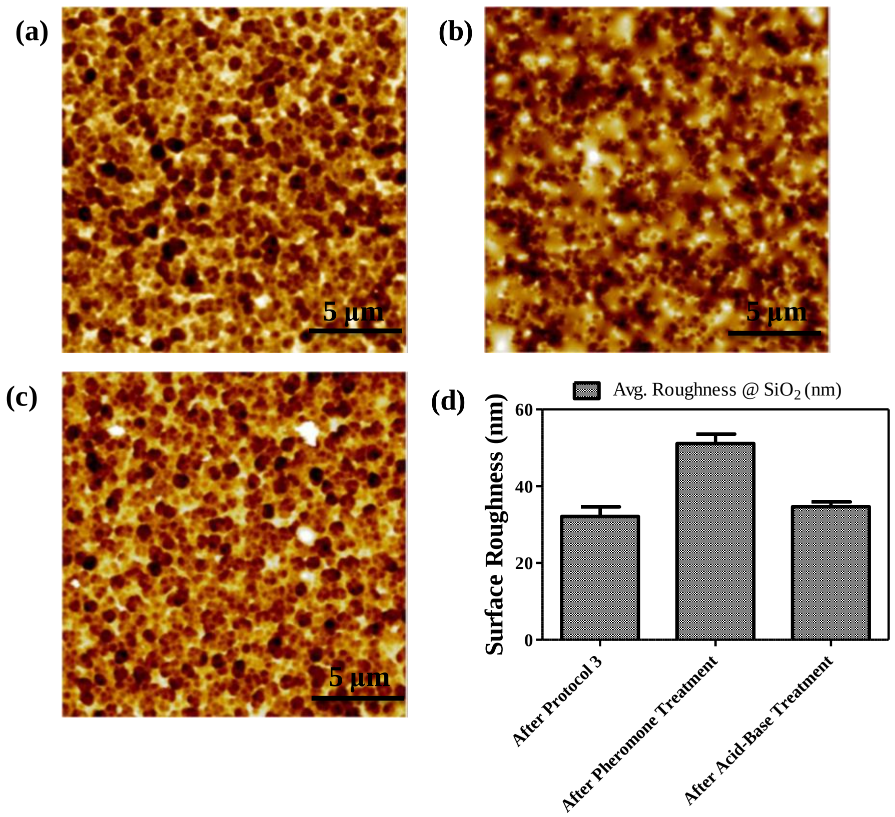

Figure S7. Reversibility of the functionalized MEMS devices: Representative AFM images of the SiO2 surfaces after (a) the functionalization by protocol 3, (b) the exposure to the pheromones and (c) the acid-base treatment of the pheromone exposed surfaces to regenerate the initial topography. The functionalized surface is then available for reversible use for further attachment with the pheromone molecules. The relative surface roughness values are depicted in (d).

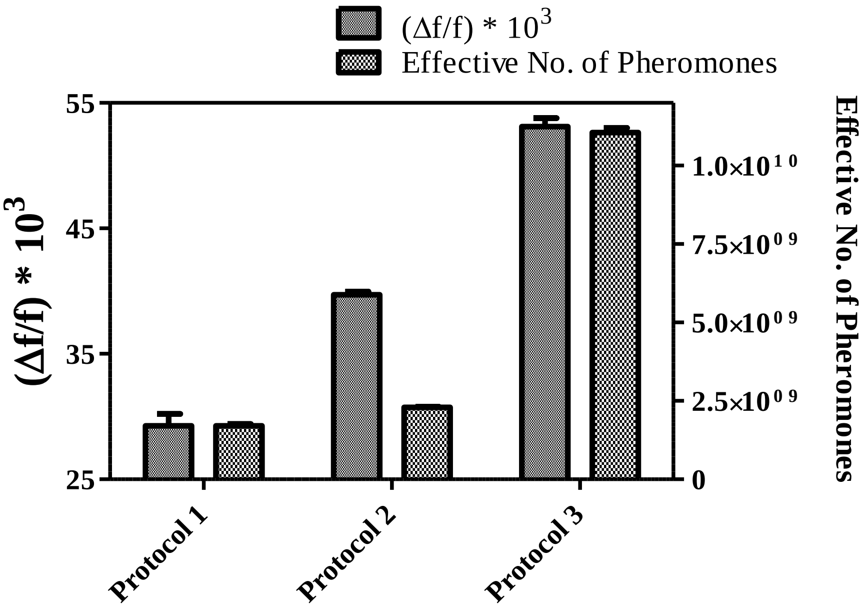

Figure S8. Comparison among the protocols for sensing of insect pheromone: Superior response of the cantilever having the length of 36.53 μm functionalized by the protocol 3 in contrast to the protocols 1 and 2. The comparison was made with Δf/f and the effective number of pheromone molecules attached on to the silicon dioxide surface.

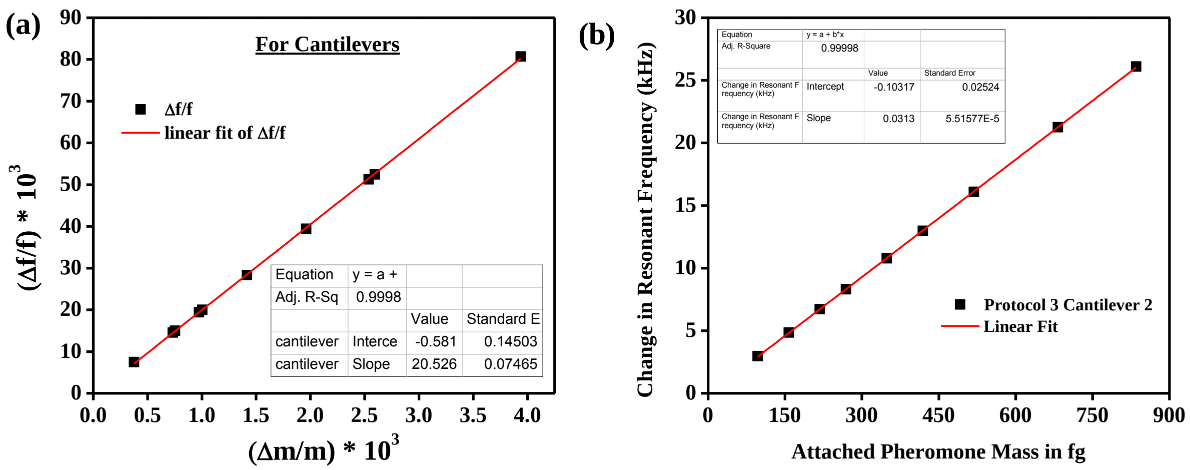

Figure S9. (a) Plot of Δf/f vs. Δm/m for the cantilevers functionalized by the protocol 3 and (b) a determination of the limit of detection and the limit of quantification for this particular cantilever.

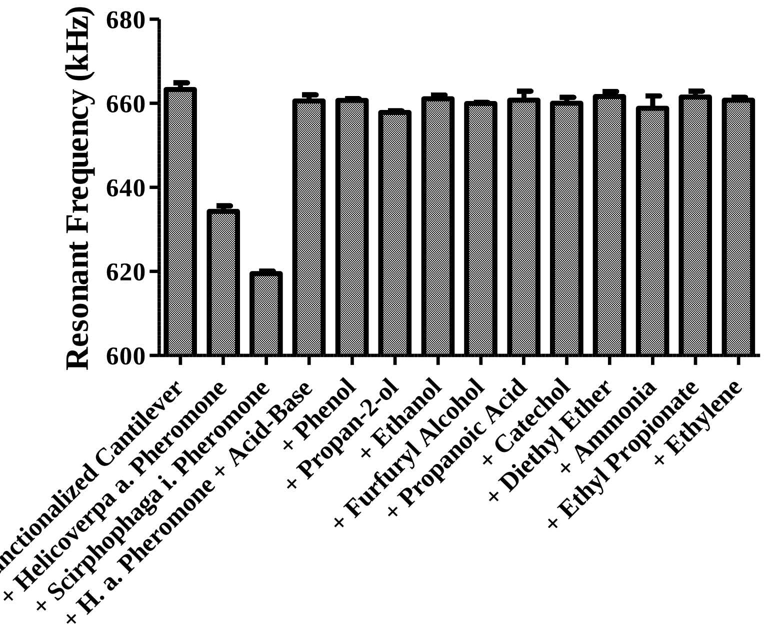

Figure S10. Selectivity and reversibility of a particular cantilever having the length of 36.53 μm functionalized by the protocol 3 in the presence of various interfering semiochemicals. The reversibility was achieved by the simple acid-base treatment.

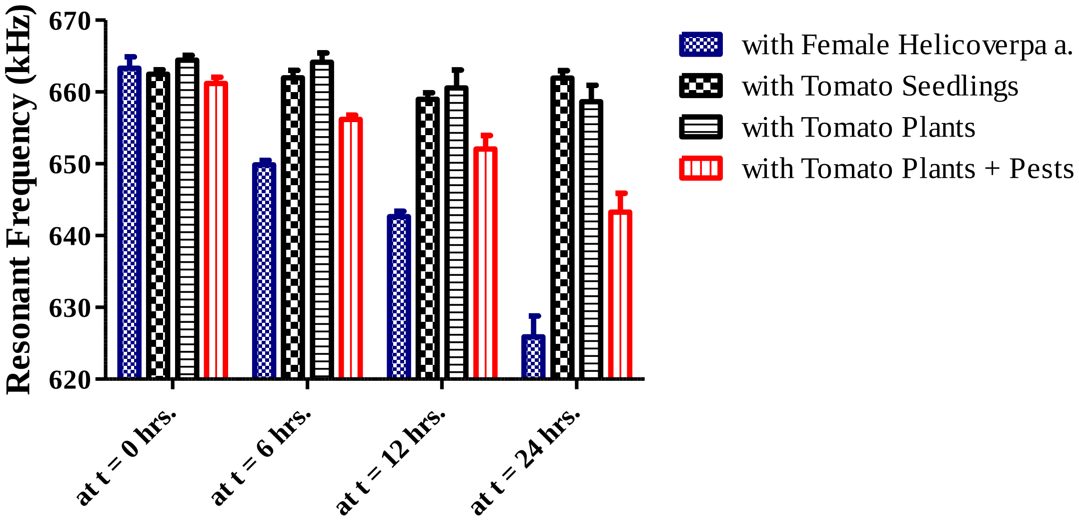

Figure S11. Data with live insects at field prototype conditions: Change in the first order resonant frequency of the cantilever functionalized by the protocol 3 having the length of 36.53 μm in the presence of female *Helicoverpa armigera* insects, tomato seedlings and tomato plants. Data were also represented when the tomato plants were infested with both the male and female insects.
